# The Complement System in ANCA‐Associated Vasculitis: Mechanistic Insights, Therapeutic Horizons, and Unmet Clinical Needs

**DOI:** 10.1002/iid3.70338

**Published:** 2026-02-12

**Authors:** Kehinde Sunmboye, Pauline Millan

**Affiliations:** ^1^ Rheumatology University Hospitals of Leicester NHS Trust, Leicester, GBR Leicester England UK; ^2^ College of life sciences, University of Leicester Leicester England UK

**Keywords:** alternative pathway, ANCA‐associated vasculitis, C5a receptor antagonists, complement activation, complement targeted therapy

## Abstract

**Background:**

ANCA‐associated vasculitides (AAV) are autoimmune small‐vessel vasculitides characterized by necrotizing inflammation with few immune deposits (“pauci‐immune” lesions). Historically, complement was thought to play a minor role in AAV due to scant complement deposition on biopsy. However, growing evidence from animal models and patient studies indicate that complement activation, particularly the alternative pathway, is a critical amplifier of inflammation in AAV.

**Methods:**

We searched PubMed, Embase, and Web of Science (January 2000–October 2024) for peer‐reviewed studies on complement in AAV prioritizing preclinical, clinical, and therapeutic research.

**Findings:**

Complement activation bridges innate and adaptive immunity in AAV: ANCA‐activated neutrophils release factors that trigger the alternative complement pathway, generating C5a, which further recruits and primes neutrophils, creating a self‐amplifying loop of inflammation. Complement components (C3a, C5a, C5b‐9) are detectable in active AAV patients' plasma/urine and in affected tissues, correlating with disease activity and severity. Therapeutically, the C5a‐receptor antagonist, avacopan has demonstrated clinical efficacy, achieving remission with reduced corticosteroid exposure.

**Conclusion:**

Complement dysregulation is a pivotal mechanism in AAV pathogenesis, opening a new era of complement‐targeted therapies. Avacopan's success illustrates the potential to improve outcomes and safety by modulating complement. Yet, major unmet clinical needs remain. Up to 50% of patients relapse within 5 years. There are no validated biomarkers to predict relapse. Long‐term steroid dependence and cumulative toxicity from rituximab and cyclophosphamide compromise patient safety. Better tools are needed to detect early disease, stratify relapse risk, and personalize therapy. Future directions must prioritize biomarker development, novel complement inhibitors, and deliver durable remission with fewer side effects.

## Graphical abstract

Complement activation, particularly via the alternative pathway, drives inflammation and organ damage in ANCA‐associated vasculitis. This review highlights mechanistic insights, tissue and biomarker evidence, and clinical implications of targeting the C5a–C5aR1 axis. Emerging therapies such as avacopan offer steroid‐sparing efficacy and represent a shift toward mechanism‐based vasculitis treatment.

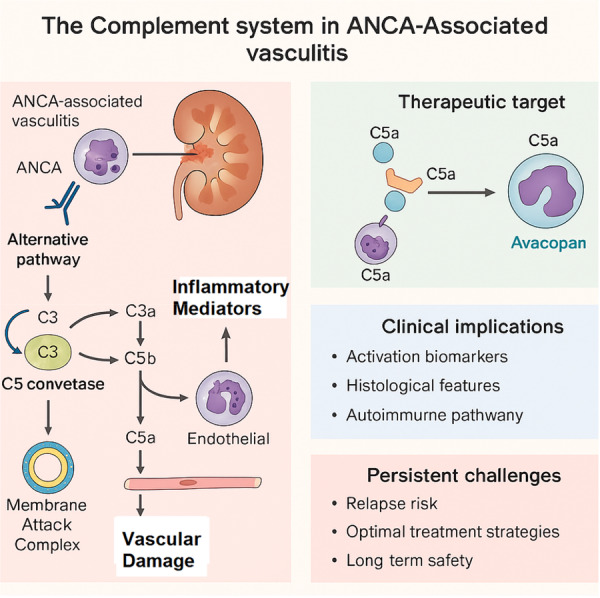

## 1 Introduction

Antineutrophil cytoplasmic antibody (ANCA)‐associated vasculitis (AAV) is a group of rare, severe autoimmune diseases defined by inflammation and necrosis of small blood vessels, usually accompanied by circulating ANCAs [1]. AAV encompasses granulomatosis with polyangiitis (GPA), microscopic polyangiitis (MPA), eosinophilic GPA (EGPA), and renal‐limited pauci‐immune glomerulonephritis. These diseases typically present with life‐threatening organ involvement (e.g., rapidly progressive glomerulonephritis and pulmonary capillaritis) and, if untreated, have high mortality [2]. The term “pauci‐immune” reflects the classic finding of little or no immunoglobulin and complement deposition on affected tissues [3]. In contrast to immune‐complex vasculitides (like IgA vasculitis or cryoglobulinemic vasculitis) where abundant immune deposits and complement activation are evident, AAV lesions have scant immunoreactants. This led to an early erroneous assumption that complement was not a major driver of AAV pathogenesis [4].

Despite the paucity of deposits, a resurgence of research over the past two decades has firmly implicated the complement system in AAV. Pioneering animal studies showed that blocking complement (specifically the C5a–C5a receptor axis) protects against ANCA‐induced vasculitis [5]: See Figure 4]. Subsequent clinical observations revealed that a subset of AAV patients have hypocomplementemia (particularly low C3) during active disease, and that complement activation products are elevated in plasma and urine during flares [6]. Moreover, detailed biopsy analyses demonstrate deposition of complement components (C3d, C5b‐9, factor B, properdin) in renal lesions of AAV, correlating with severe histologic damage [7, 8]. These findings overturned earlier dogma and established the complement cascade, especially the alternative pathway as a critical mediator in AAV [9].

Against this backdrop, this review will summarize complement system biology relevant to AAV, examine mechanistic insights into how complement fuels AAV pathogenesis, review current and emerging complement‐targeted therapies (highlighting the novel agent avacopan), and explore future directions and ongoing clinical unmet needs and challenges for patients in the quest for more precise, safe, and effective treatments for AAV. By looking into these areas, we aim to clearly communicate how the complement system underpins disease pathogenesis and therapy in AAV, while also noting broader implications for the unmet clinical needs that remain in patients with AAV [10].

## 2 Methods

This review provides a critical synthesis of mechanistic insights, therapeutic innovations, and persistent clinical challenges related to complement activation in ANCA‐associated vasculitis (AAV). We conducted a structured narrative review, selecting studies that advance understanding across immunopathogenesis, biomarker development, therapeutic application and unmet patient needs in AAV.

**Figure 1:**
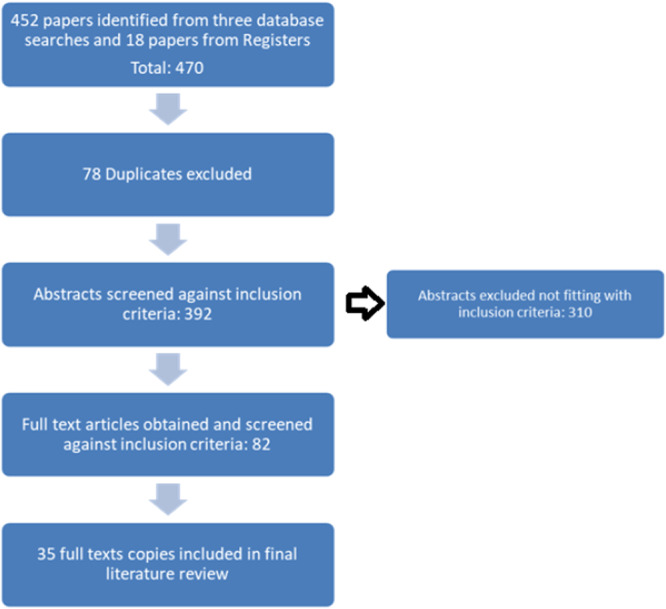
Literature search flow chart: Adapted from PRISMA (Mohler et al., 2009).

We systematically searched PubMed, Embase, and Web of Science for English‐language, peer‐reviewed publications from January 2000 to October 2024. Search terms included “ANCA‐associated vasculitis,” “complement system,” “AAV,” and “complement inhibitor therapy.” studies were included (see Figure 1) if they contributed original data on complement‐mediated mechanisms, clinical phenotyping, or treatment interventions in AAV. We focused on preclinical investigations that clarified pathogenic pathways, clinical cohorts evaluating complement biomarkers or outcomes, interventional trials assessing complement‐targeted therapeutics and unmet clinical needs.

Exclusion criteria for the review includes case reports, conference abstracts, and studies lacking primary data. Randomized controlled trials, translational studies, and well‐designed mechanistic experiments were prioritized. Each study underwent critical appraisal with attention to internal validity, sample size, reproducibility, and sources of bias particularly assessing in greater detail industry funding and obvious conflicts of interest.

Data extraction was guided by a thematic framework to examine: how complement dysregulation drives inflammation and tissue damage in AAV; the clinical efficacy and safety of agents like avacopan targeting the C5a‐C5aR axis; and unmet clinical needs in disease monitoring, relapse prediction, and individualized therapy. Our thesis aims to inform future research directions, therapeutic strategies, and biomarker development to address the ongoing burden of relapse, toxicity, and suboptimal disease control in this complex and continuously evolving disease area.

### 2.1 Complement System Biology

**Figure 2:**
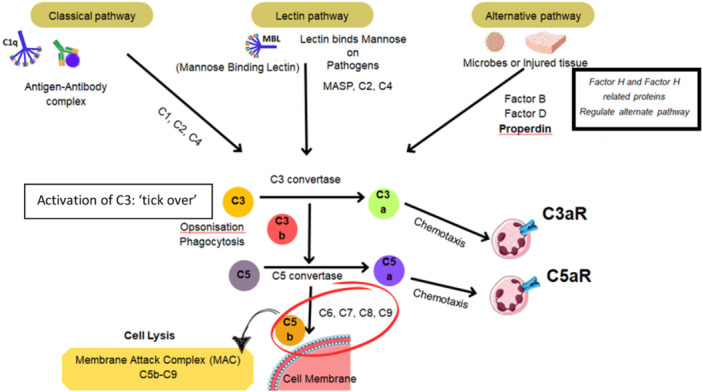
Complement system highlighting alternative pathway with the red ellipse showing components of the membrane attack complex (MAC), C5b‐C9.

The complement system (Figure 2) is a cornerstone of innate immunity, consisting of a cascade of plasma proteins that, once activated, enhance phagocytosis, cell lysis, and inflammation [11]. Complement can be activated via three pathways: classical, lectin, and alternative pathway which converge to a common terminal pathway. The classical pathway is triggered by antibodies (immune complexes) or certain pentraxins binding to C1q, leading to sequential activation of C1r/s, C4, and C2, and formation of the C3 convertase C4b2a. The lectin pathway is activated by pattern‐recognition molecules like mannose‐binding lectin (MBL) or ficolins binding to microbial carbohydrate motifs or damaged‐cell glycans, which then activate MASP‐1/2 to cleave C4 and C2 (forming the same C4b2a convertase).

In contrast, the alternative pathway is constitutively poised for activation. C3 hydrolysis generates C3b that can attach to cell surfaces. If not regulated, C3b binds factor B, which factor D then cleaves to form the C3 convertase C3bBb; this is stabilized by properdin on microbial or damaged surfaces [12]. All pathways generate C3b and then assemble C5 convertases (C4b2a3b for classical/lectin, or C3bBbC3b for alternative). These enzymes cleave C5 into C5a and C5b. C5b initiates formation of the membrane attack complex (MAC: C5b‐9) which can directly lyse cells or induce cell injury. Meanwhile, the small fragments C3a and C5a (anaphylatoxins) potently chemoattract and activate leukocytes, induce reactive oxidative bursts, increase vascular permeability, and amplify inflammation [13] Table 1.

**Table 1:** Complement pathways and effector components implicated in ANCA‐associated vasculitis (AAV): Complement activation in AAV primarily involves the alternative pathway, with contributions from classical and lectin pathways. Key mediators such as C3b, C5a, and the membrane attack complex (MAC) drive inflammation, while dysregulation of complement control proteins may amplify tissue damage.

Complement component or pathway	Role and relevance to AAV
C3 (central complement protein)	Cleaved into C3b and C3a upon activation. C3b opsonizes surfaces and amplifies the cascade; C3a is an anaphylatoxin causing leukocyte recruitment. In AAV, C3 fragments (C3c, C3d) are found in glomeruli, and low serum C3 can indicate active disease [8].
Alternative pathway (AP)	Continuously active at low level; major amplification loop for complement. AP components (factor B, properdin) are detected in AAV kidneys. AP activation generates C3b and C5a, fueling neutrophil activation. Inhibition of AP (e.g., in animal models) protects against pauci‐immune vasculitis [5].
Classical pathway (CP)	Triggered by immune complexes binding C1q. Historically deemed less relevant in AAV due to “pauci‐immune” nature. However, immune complexes and C1q deposition are observed in some AAV patients, suggesting CP contributes in certain cases (notably PR3‐ANCA disease) [26].
Lectin pathway (LP)	Initiated by pattern recognition (e.g., MBL, ficolins) binding to microbes or altered self. Emerging evidence hints LP involvement in AAV: for example, higher MBL levels correlate with disease activity in AAV. LP may be more relevant in related IgA vasculitis, but AAV cases with infections could engage LP [29].
C5a (anaphylatoxin)	Potent chemoattractant and neutrophil activator via the C5a receptor (C5aR/CD88). C5a levels are elevated in active AAV plasma. C5a is a central mediator linking complement to ANCA‐induced neutrophil damage. Blocking C5a or C5aR abrogates disease in animal models and is effective therapeutically (e.g., avacopan) [24].
Membrane attack complex (MAC)	C5b‐9 complex that can lyse cells or induce inflammation. Detected in kidneys of AAV patients as C5b‐9 deposits. Likely contributes to endothelial injury in glomeruli and perhaps vascular necrosis in severe disease. Elevated circulating sC5b‐9 in active AAV reflects ongoing complement terminal pathway activation [7].
Complement regulators (e.g., Factor H, Factor H‐Related proteins, C1‐INH)	Limit complement activation on host tissues. In AAV, an imbalance or local dysfunction of regulators may occur in inflamed sites. For instance, Factor H‐Related Protein 1 (FHR1) can bind necrotic cells and promote complement activation; its presence marks necrotic vasculitic lesions. Genetic absence of FHR1 (common in some populations) and anti‐complement regulator autoantibodies are areas of ongoing research in AAV pathogenesis [21].

**Figure 3:**
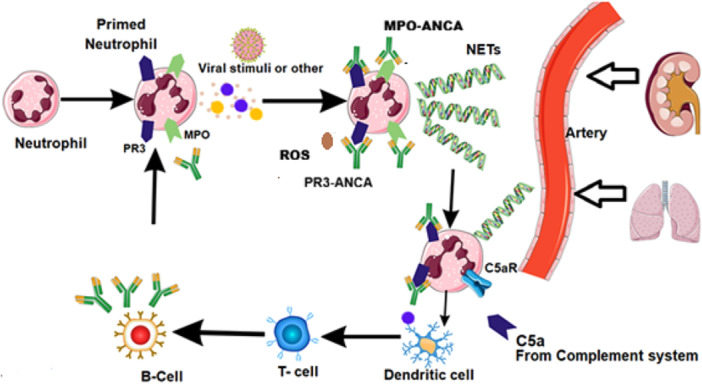
Pathogenic mechanisms linking neutrophil priming, ANCA activation, and complement amplification in ANCA‐associated vasculitis (AAV). ROS: Reactive oxygen Species. Note that activated neutrophils can migrate and extravasate out of the damaged vessel.

Tight regulation by complement inhibitors (such as factor H, factor I, C1‐inhibitor, CD55, CD59, etc.) normally prevents excessive complement activation on host tissues [14]. In health, this balance allows complement to attack microbes or clear apoptotic cells without injuring self. In pathological settings, however, dysregulated complement activation can cause host tissue damage (Figure 3). For instance, inherited or acquired defects in complement regulators underlie diseases like atypical hemolytic uremic syndrome and C3 glomerulopathies, wherein uncontrolled complement harms endothelial cells and glomeruli [15]. In vasculitis, even in the absence of genetic mutations, intense inflammation can tip the balance toward complement activation on endothelium and circulating cells.

2.2

#### 2.2.1 Pathogenic Role of Complement in AAV

##### 2.2.1.1 Mechanistic Insights: ANCA, Neutrophils, and the Complement Loop

AAV pathogenesis is initiated by ANCAs targeting neutrophil antigens (primarily myeloperoxidase [MPO] or proteinase‐3 [PR3]) [16]. When neutrophils are “primed” (Figure 3) (e.g., by cytokines or microbial products) to express MPO/PR3 on their surface, ANCAs can bind and activate these leukocytes [17]. Activated neutrophils adhere to endothelium and release lytic enzymes, reactive oxygen species, and form neutrophil extracellular traps (NETs), causing focal necrosis of vessel walls [18]. This neutrophil‐driven damage is the hallmark of AAV, but complement greatly amplifies this destructive process [19].

An elegant self‐perpetuating loop has been elucidated: ANCA‐activated neutrophils themselves spark complement activation, which in turn recruits and activates more neutrophils (Figure 3) [20]. Specifically, stimulated neutrophils release cationic enzymes (e.g., elastase, MPO) that can consume local endothelial protective factors and expose basement membrane surfaces that lack complement regulation, favoring alternative pathway activation [21]. Neutrophils also exocytose properdin, the only positive regulator of complement, which can bind to damaged tissues or NETs and stabilize C3 convertases [14]. The result is a burst of complement activation with generation of C3a and C5a. C5a is a potent neutrophil chemoattractant and activator: it binds C5a receptors on neutrophils and monocytes, lowering the threshold for their activation and prompting the release of more inflammatory mediators [13]. C5a not only draws additional neutrophils into tissues, but “primes” them upregulating adhesion molecules and causing mobilization of more ANCA antigens to the cell surface [16]. Thus, C5a dramatically enhances ANCA‐induced neutrophil activation. In essence, ANCA and C5a synergize to convert circulating neutrophils into destructive effector cells, establishing a vicious cycle of neutrophil recruitment, activation, and further complement activation [22].

**Figure 4:**
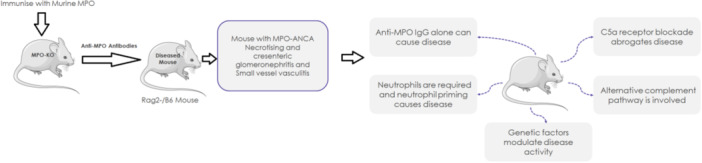
Lessons from the Mouse Model on the Role of the Complement System in AAV. In this model, mice immunized with murine myeloperoxidase (MPO) produce anti‐MPO antibodies that induce necrotizing crescentic glomerulonephritis and small vessel vasculitis when transferred into Rag2⁻/⁻ B6 mice. The findings show that anti‐MPO IgG alone can cause disease, but neutrophil priming and recruitment are essential for disease expression. Genetic background influences disease severity. Crucially, C5a receptor blockade abrogates disease, implicating the alternative complement pathway as a key driver of MPO‐ANCA‐associated vasculitis.

Neutrophil extracellular traps provide another mechanistic link between ANCA and complement. NETs are web‐like structures of decondensed chromatin decorated with granular proteins (MPO, PR3) and complement components [20]. In AAV, NETs have two pathogenic consequences: they serve as a source of autoantigen that may stimulate ANCA production, and they provide a surface for alternative and lectin pathway complement activation [21]. NETs can bind C1q and MBL, potentially activating the classical and lectin pathways, respectively, in addition to amplifying the alternative pathway [23]. Indeed, studies have detected complement components co‐localized on NETs within AAV lesions [20]. The NETs also fail to be adequately cleared in AAV (possibly due to impaired DNases or overwhelming production), prolonging their pro‐inflammatory effects [18]. Altogether, the neutrophil‐ANCA‐complement interaction forms a feed‐forward loop: ANCA activates neutrophils; neutrophils activate complement; complement (C5a) powerfully recruits and activates more neutrophils perpetuating inflammation and tissue damage [20] Figure 4.

Crucial evidence for this loop comes from animal models. In a landmark study, mice injected with anti‐MPO antibodies (to induce an MPO‐ANCA vasculitis model) were protected from developing glomerulonephritis if they were genetically deficient in C5 or the C5a receptor (C5aR) [5]. Similarly, pharmacologic blockade of C5aR in mice markedly attenuated neutrophil infiltration and capillary injury in experimental AAV [24]. These experiments demonstrated that ANCA alone is not sufficient to cause full‐blown vasculitis without complement activation. C5a‐C5aR signaling was required to unleash neutrophil‐mediated vessel damage [24]. Additional studies confirmed that inhibition at different complement points can ameliorate disease in vivo: for example, an inhibitor of human C5aR (CCX168, later known as avacopan) reduced glomerulonephritis severity in rodent models, and C4 or factor B knockout mice also showed resistance to ANCA injury in some models [5]. Taken together, these data firmly establish complement, especially the C5a–C5aR axis, as a lynchpin in AAV pathogenesis [22].

It is important to note that while the alternative pathway provides the major amplification in AAV, the classical and lectin pathways may also contribute upstream [25]. ANCAs are IgG antibodies; although they typically cause direct neutrophil activation rather than forming large circulating immune complexes, small immune complexes could form and deposit in tissues in certain circumstances [26]. Indeed, up to 30%–60% of AAV patients have detectable levels of circulating immune complexes or low levels of Immunoglobulin deposition in kidneys [27]. One study found that 65% of AAV patients had positive tests for circulating immune complexes by a rheumatoid factor‐based assay [28]. Histologically, C1q deposition in renal biopsies of AAV is “not uncommon”, though usually less intense than in immune‐complex glomerulonephritis [26]. These findings suggest that the classical pathway can be engaged in AAV, particularly in MPO‐ANCA disease or in severe inflammation where secondary immune complexes form [25]. A recent analysis by Wu et al. [25] even indicated that PR3‐ANCA patients might exhibit more classical/lectin pathway activation (e.g., higher C4d levels) compared to MPO‐ANCA patients. The lectin pathway may also be invoked by exposure of abnormal glycans on damaged endothelium or microbes: for instance, higher plasma MBL levels were associated with AAV disease activity and lung hemorrhage in one cohort [29]. Thus, while alternative pathway amplification is central, upstream triggers via classical or lectin routes likely provide the sparks that ignite complement activation in at least some AAV contexts [19].

##### 2.2.1.2 Histological and Clinical Evidence of Complement Activation

Direct evidence of complement activation in AAV comes from renal histopathology and clinical biomarkers. Although AAV is defined by “pauci‐immune” histology, careful immunohistochemistry often detects complement fragments in affected tissues [7]. In renal‐biopsy studies of pauci‐immune necrotizing glomerulonephritis, C3 deposits are observed in a substantial subset of patients [8]. For example, in a cohort of 187 AAV patients, 42% had C3c deposition on immunofluorescence [8]. MPO‐ANCA patients showed a higher frequency of C3 deposition than PR3‐ANCA patients (52% vs. 32% in that series), aligning with clinical observations that hypocomplementemia tends to be more frequent in MPO‐ANCA disease [28]. C3d (the degradation product of C3) was positive in about fifty to seventy percent of cases, indicating complement activation had occurred in those glomeruli [7]. Notably, cases with C3d and properdin deposition had significantly more glomerular crescents and fewer normal glomeruli than those without such deposits, linking complement deposition to more aggressive pathology [8]. Similarly, staining for the membrane attack complex (C5b‐9) and factor B in renal tissue has been reported, particularly in active lesions with necrosis [7]. Properdin, when present in biopsies, tends to localize in areas of crescent formation, consistent with alternative pathway amplification right at the sites of severe injury [21].

Interestingly, one study found C4d (a marker of classical/lectin pathway activity) in ~71%–88% of AAV kidney biopsies, despite only a minority having significant Immunoglobulin deposits or C1q [30]. This suggests that even in “pauci‐immune” AAV, complement can be activated through pathways other than the alternative route, possibly by low levels of immune complexes or lectin pathway triggers [25]. Electron microscopy in some AAV biopsies has revealed small electron‐dense immune deposits in the glomerular basement membrane (in about 7% of cases), corroborating that immune complexes may play a role in a subset of patients (sometimes described as “ANCA plus immune‐complex” glomerulonephritis) [26]. These cases often have more pronounced complement activation and may portend worse renal outcomes [27].

Beyond tissue deposition, circulating complement factors reflect activation in vivo. Patients with active AAV frequently show consumption of complement components and elevation of split products [7]. Low serum C3 at presentation has been associated with more severe disease and worse outcomes [6]. For instance, a 2015 study reported that AAV patients with hypocomplementemia (especially low C3) had higher risk of end‐stage renal disease and death; low C3 also correlated with renal histologic signs of thrombotic microangiopathy (TMA) in that cohort [6]. TMA lesions (fibrin microthrombi in glomeruli or arterioles) are found in a subset of AAV biopsies and may result from severe complement‐mediated endothelial injury [8]. The presence of TMA in AAV has been linked to complement activation and may identify a particularly aggressive phenotype; indeed, there are reports of AAV patients with TMA benefiting from complement inhibitors (like eculizumab) in refractory situations [6].

Multiple studies have quantified circulating complement activation products in AAV. Plasma levels of C3a, C5a, and soluble C5b‐9 (sMAC) are elevated in active AAV compared to healthy controls [7]. In one prospective study, active AAV patients had significantly higher median concentrations of C5a and sC5b‐9 than those in remission or in healthy individuals [25]. Factor B levels were also higher in active disease, while properdin levels paradoxically were often lower during flares (likely consumed due to ongoing alternative pathway turnover) [7]. Upon remission (after immunosuppressive therapy), C5a and C3a levels tend to decrease, reflecting dampened complement activation [31]. A meta‐analysis of published data confirmed that active AAV is associated with higher C5a, sC5b‐9, and factor B levels than inactive disease [9]. These biomarkers imply that complement activation rises and falls with disease activity [31].

Analysis of patient urine samples provides another window into organ‐specific complement activity. In pauci‐immune crescentic GN, complement breakdown products can be detected in urine and often mirror intrarenal activation. Shen‐Ju Gou and colleagues demonstrated that urinary levels of Bb, C3a, C5a, and C5b‐9 are markedly elevated in patients with active ANCA GN compared to those in remission or to other renal diseases [7]. Importantly, urinary complement levels declined following effective immunosuppressive treatment, suggesting they could serve as non‐invasive markers of renal disease activity [31]. Taken together, the presence of complement fragments in blood and urine, along with renal biopsy evidence, paints a consistent picture: complement is activated during AAV flares and contributes to the immunopathology [32].

#### 2.2.2 Diagnostic and Prognostic Implications

##### 2.2.2.1 Biomarkers of Complement Activation in AAV

The recognition of complement's role in AAV has spurred interest in complement biomarkers for disease monitoring and prognostication [31]. Traditional biomarkers in AAV include ANCA titers, acute‐phase reactants, and urinary findings, but these are imperfect predictors of flare or outcome [33, 34]. Complement activation products offer an additional dimension reflecting the innate immune status. As noted, low serum complement levels (especially C3) at diagnosis can indicate a high‐risk patient [6]. For example, Manenti et al. [6] found that AAV patients with low C3 had higher rates of kidney failure and mortality. Low C3 might result from consumption by ongoing complement activation or concurrent TMA, and thus serves as a red flag for aggressive disease [8]. In routine practice, most AAV patients have normal complement levels, but checking C3 and C4 at baseline is useful: if C3 is depressed, closer monitoring or more intensive therapy may be warranted [6]. (Low C4 is less common in AAV, aligning with alternative pathway predominance, whereas a very low C4 might prompt a search for an immune‐complex process or cryoglobulinemia overlap [35]).

Dynamic changes in complement fragments have potential as activity biomarkers. Research assays (ELISA or mass spectrometry‐based) can measure plasma or urinary C5a, C3a, Ba, Bb, and sC5b‐9 [31]. In general, active AAV shows higher levels of these fragments, which decrease with remission [7]. One study of paired samples showed complement activation markers peaking at relapse and falling after treatment, correlating with the Birmingham Vasculitis Activity Score (BVAS) [25]. Urinary C5b‐9 in particular has been proposed as a marker of active glomerulonephritis, as it directly reflects complement attack within the kidneys [31]. These assays are not yet standard in clinical practice, but they hold promise. In the future, a “complement panel” could potentially augment clinical and serologic assessment to confirm a flare or gauge response to therapy [31]. For instance, a patient with rising ANCA titer and vague symptoms could be assessed for a concurrent rise in urinary Bb or plasma C5a, which if present might increase confidence that a true relapse is occurring that merits treatment [31].

An intriguing application of complement biomarkers is in guiding therapy choice. Since complement inhibition therapy (such as avacopan) is now available, one might ask: can we identify patients who will benefit most from it? [36]. It is plausible that patients with evidence of high complement pathway activation (e.g., low C3, high sC5b‐9, or strong C3d deposition on biopsy) might derive the greatest benefit from adjunctive complement inhibition [37]. A recent review suggested that assessing complement activation could help pinpoint patients for complement‐targeted therapy [22]. However, this approach remains to be tested. At present, there is no validated cutoff of a complement marker that predicts avacopan responsiveness [31]. Nonetheless, ongoing studies are collecting biosamples to correlate complement profiles with outcomes in treated patients. In time, we may incorporate complement biomarkers into personalized treatment algorithms which will be an example of the precision medicine approach in vasculitis.

##### 2.2.2.2 Therapeutic Developments: Complement‐Targeted Interventions

The growing understanding of complement's role in AAV has directly informed new therapeutic strategies [22]. For decades, AAV treatment has relied on non‐specific immunosuppression: high‐dose glucocorticoids (GCs) combined with cyclophosphamide or rituximab for induction, followed by lower‐intensity maintenance [38, 39]. While effective, this approach carries significant toxicity (particularly from chronic steroids and cyclophosphamide) [40]. The identification of C5a as a key driver of neutrophil‐mediated damage provided a target for a more specific intervention, namely, blocking the C5a–C5aR axis [24]. This has led to the development of Avacopan, an orally active C5a receptor antagonist, and its introduction into clinical practice marks a milestone in AAV therapy [41].

### 2.3 Avacopan (C5aR Inhibitor)

Avacopan was the first complement‐targeted drug approved for AAV [42]. Avacopan efficacy was demonstrated in a human C5aR knock‐in murine model, as the drug does not bind murine C5aR [41]. In the pivotal Phase 3 ADVOCATE trial, avacopan (in combination with standard cyclophosphamide or rituximab regimens) was compared to a standard prednisone‐taper regimen (plus cyclophosphamide or rituximab) in patients with acute GPA or MPA [41]. Avacopan allowed for elimination of daily oral steroids after a very brief initial pulse; essentially, it was a steroid‐sparing strategy. The results were ground‐breaking: avacopan was non‐inferior to standard care for inducing remission at 26 weeks, with ~72.3% of the avacopan group versus ~70.1% of the prednisone group achieving remission [41]. Importantly, at 52 weeks, avacopan was superior in sustaining remission: 65.7% of avacopan‐treated patients maintained remission versus 54.9% on the prednisone‐based regimen [41]. This showed that not only could avacopan allow for steroid reduction without loss of efficacy, it might confer more durable disease control [37]. Furthermore, patients on avacopan had greater improvement in kidney function with the mean eGFR rise at 1 year of +7.3 mL/min in the avacopan group versus +4.1 mL/min in the prednisone group [41]. This suggests that avacopan may directly protect the kidney (perhaps by preventing ongoing C5a‐driven injury during repair). Clinically, avacopan recipients reported better quality of life and fewer steroid‐related side effects (such as hyperglycemia, weight gain, mood disturbance) [41]. Based on these findings, avacopan (now marketed as Tavneos) received regulatory approvals (FDA in 2021, EMA in 2022) for use in GPA/MPA in combination with standard immunosuppressive therapy as a means to reduce glucocorticoid requirements.

It should be noted that avacopan is not a stand‐alone treatment. In trials it was used with either rituximab or cyclophosphamide. It replaces the corticosteroid component of induction remission therapy to reduce steroid exposure. In clinical practice, avacopan is typically started at induction and continued for 12 months (it was given for 52 weeks in the trial) in concert with the immunosuppressive drug [37]. Avacopan's advent has been met with excitement but also practical considerations: it is expensive, and long‐term safety beyond 1 year remains under observation [42]. Nonetheless, it represents a proof of concept that targeting complement can control AAV [22]. Many patients who cannot tolerate high‐dose steroids (e.g., due to diabetes, osteoporosis, or psychosis) may particularly benefit from avacopan. The 2022 updated EULAR recommendations for managing AAV endorse avacopan as part of a strategy to minimize corticosteroid exposure in GPA/MPA.

#### 2.3.1 Other Complement‐Targeted Therapies

Beyond avacopan, several other approaches to complement inhibition are in development for AAV.

#### 2.3.2 C5a Monoclonal Antibody (Vilobelimab)

Vilobelimab (also known as IFX‐1) is an antibody that neutralizes C5a (preventing it from engaging C5aR). Unlike Avacopan, which blocks the receptor, vilobelimab soaks up the ligand [43]. A Phase 2 trial in Europe (IXCHANGE) explored vilobelimab in GPA/MPA. Patients received standard cyclophosphamide or rituximab and were randomized to either vilobelimab with reduced‐dose steroids, vilobelimab without steroids, or standard‐dose steroids (control). The trial found that vilobelimab was well tolerated and appeared to yield remission rates comparable to the steroid‐containing regimen, although the study was not powered for definitive efficacy conclusions [unpublished data, 2022]. A parallel U.S. Phase 2 (IXPLORE) also indicated that C5a blockade is safe and can significantly spare steroid use [unpublished data, 2022]. These preliminary results are encouraging suggesting that C5a neutralization, like C5aR blockade, can control disease [43]. Ongoing larger studies will determine if anti‐C5a therapy can achieve regulatory approval. One theoretical advantage of ligand blockade is avoiding any potential agonist effect at the receptor (though avacopan has not shown such issues) [41]. A potential disadvantage is that very high C5a levels (in explosive complement activation) might be harder to neutralize completely [43]. There is uncertainty about the role of C5aR2, the other receptor of C5a, which may be inhibitory or activatory and that no further study has been planned thus far.

#### 2.3.3 C5 Inhibitors (Eculizumab, Others)

Eculizumab, a monoclonal antibody against C5, is a broad complement inhibitor that prevents generation of both C5a and C5b‐9 [44]. It is approved for atypical HUS, anti‐C5 nephritis, and other complement‐driven diseases [45]. While not trialed in AAV, eculizumab has been used off‐label in a few refractory cases [6]. For instance, case reports document that eculizumab helped halt rapidly progressive vasculitis in patients who had severe AAV with concurrent TMA or those who failed standard therapy [44]. In one report of three patients with refractory AAV, eculizumab led to improved renal function and disease remission without major infectious complications [46]. However, using eculizumab in AAV carries a high risk of infection (especially meningococcal disease due to loss of MAC function) and thus requires prophylactic antibiotics/vaccination [15]. Because AAV can often be controlled with less extreme immunotherapy, eculizumab is generally reserved for extraordinary circumstances such as a patient with concomitant aHUS or catastrophic anti‐GBM disease overlapping with AAV [6]. Newer long‐acting C5 inhibitors (ravulizumab, nomacopan, etc.) could theoretically be considered similarly [45]. At this time, C5 inhibition in AAV remains an anecdotal approach rather than standard of care.

#### 2.3.4 Alternative Pathway Inhibitors

Given the centrality of the alternative pathway, drugs that target upstream AP components are logical candidates. One such target is factor B, essential for the AP C3 convertase. An oral factor B inhibitor (iptacopan) has shown efficacy in C3 glomerulopathy and is being studied in other renal diseases [47]. Although no trials in AAV have been reported, iptacopan or similar agents might attenuate complement amplification in AAV [43]. Another target is factor D, the protease that activates factor B; factor D inhibitors (e.g., danicopan) are in development for PNH and could be repurposed for AAV research [43]. Properdin inhibitors could also downregulate AP activity [14]. As of 2025, these strategies are still speculative for AAV, but they represent the next wave of complement‐directed therapy that could be tested, especially if avacopan and vilobelimab usage confirms the importance of upstream complement.

#### 2.3.5 Lectin Pathway Inhibitors

If ongoing research identifies a subset of AAV where lectin pathway is pathogenic (e.g., patients with high MBL or MASP activity), one might consider lectin pathway blockade [29]. Narsoplimab, an anti‐MASP‐2 antibody, is in trials for IgA nephropathy and could conceivably find a niche in vasculitis with lectin pathway activation. Again, this is a future consideration pending more data [43].

#### 2.3.6 Plasma Exchange (PLEX)

While not a drug, it's worth mentioning plasma exchange as a historical strategy to remove circulating factors including ANCA and complement components [39]. Prior to targeted complement therapeutics, PLEX was used in severe AAV (especially with pulmonary hemorrhage or severe renal failure) to rapidly clear ANCA and possibly pro‐inflammatory mediators like C5a [41]. The PEXIVAS trial, however, showed that PLEX did not significantly improve outcomes in most AAV patients aside from possibly those with the most severe lung hemorrhage [48]. PEXIVAS also used aggressive immunosuppression and shorter steroid courses, which might overshadow any benefit of PLEX [48]. With agents like avacopan now available to curb complement, the role of PLEX may further diminish except in special scenarios (e.g., combined anti‐GBM disease or catastrophic presentations). It is worth pointing out that while PLEX is not recommended routinely, it may have some benefits while carrying a risk of severe infections.

#### 2.3.7 Future Directions and Unmet Patient Needs

As AAV management enters the complement inhibition era, several exciting avenues and unresolved issues lie ahead. Future directions span scientific discovery, biotechnology, and clinical strategy.

#### 2.3.8 Refining Patient Selection for Complement Inhibition Therapy

One major challenge is identifying which patients will gain the most from complement‐targeted therapies. Not all AAV cases are alike PR3‐ANCA versus MPO‐ANCA disease, varying degrees of complement activation, different organ involvements [28]. For example, MPO‐ANCA patients more often have complement consumption and might particularly benefit from complement inhibition [27]. Ongoing studies are analyzing biomarkers (like baseline C5a, C3, or gene expression profiles) to predict response to avacopan or other agents [31]. Precision medicine approaches may eventually tailor therapy: a patient with high complement activation could receive an adjunctive C5a inhibitor, whereas one with predominant T‐cell or granulomatous features might need alternative interventions [49]. Developing a reliable companion diagnostic (e.g., a “complement activation signature”) is a key future goal [31].

#### 2.3.9 Long‐Term Safety and Optimization

While short‐term trials show that complement inhibitors are generally safe in AAV, long‐term safety needs monitoring [42]. Chronic suppression of C5a might slightly increase certain infection risks (though surprisingly, avacopan did not show major infection increases compared to steroids, possibly because steroids themselves predispose to infection) [41]. Nevertheless, vigilance for infections (especially encapsulated organisms) remains prudent [15]. Additionally, rare but serious events (like hepatic dysfunction noted with Avacopan in a few cases) require pharmacovigilance [42]. As more patients receive avacopan post‐approval, real‐world data will inform us of any unanticipated adverse effects or drug interactions [42]. Another issue is whether to continue complement blockade long‐term or just for induction. In ADVOCATE, avacopan was stopped at 52 weeks; some patients then relapsed later [41]. It's unknown if extended use would maintain remission or if there is an optimal stopping point [37]. Cost‐effectiveness is also a consideration as these drugs are expensive, so defining the minimal effective duration is important.

#### 2.3.10 Expanding Therapeutic Horizons

The success with C5a/C5aR encourages exploration of other targets in the inflammatory cascade of AAV [43]. Upstream complement inhibition (alternative or lectin pathway) could be tested in research settings [14]. Beyond complement, other innate immune targets (like neutrophil elastase, MPO itself, or NET formation) are being studied [20]. Combinatorial approaches might yield synergistic effects, for example a trial could consider avacopan plus a lower dose of steroid versus avacopan alone, to see if outcomes improve further or if certain manifestations (like granulomatous disease in GPA) still require some steroid [38]. Additionally, complement inhibition might be beneficial in related vasculitides: IgA vasculitis and cryoglobulinemic vasculitis are prime examples where complement plays a role [29, 35]. In refractory IgA vasculitis nephritis with severe complement deposition, one could hypothesize that a complement inhibitor (perhaps an anti‐C5 or MASP inhibitor) could ameliorate injury [50]. In cryoglobulinemic vasculitis, especially the subset with low complement and hyperactivation of classical pathway, targeting C1 or C4 or using C5 inhibitors might reduce inflammation (though plasma exchange and antiviral/immunosuppressive therapy are mainstays at present) [51, 52].

Future clinical trials might branch out to test complement inhibitors in these conditions, leveraging the lessons learned from AAV [53].

#### 2.3.11 Addressing Persistent Clinical Challenges

Despite these therapeutic advances, several clinical challenges persist in AAV. One is the high relapse rate, especially in PR3‐ANCA vasculitis [40]. Even with avacopan and rituximab, relapses can occur and require re‐treatment [54]. It's unknown whether complement inhibitors can reduce long‐term relapse rates or if maintenance therapy with such agents is feasible [54]. Another challenge is treatment of granulomatous manifestations (like orbital masses or airway stenosis in GPA) these often respond poorly to conventional therapy and it's unclear if complement plays a significant role in these mainly localized, lesions [55]. Such manifestations might need additional targeted therapy (e.g., anti‐IL‐5 for eosinophilic granulomatosis or perhaps localized interventions) [56]. Furthermore, about 10%–20% of AAV patients have suboptimal responses or refractory disease even with our best therapies [57]. Some of these refractory cases may benefit from complement add‐on therapy (if not already given), but others might involve completely different pathways (like autoinflammatory loops or ANCA‐independent neutrophil activation) [20].

Ongoing research is probing the role of the coagulation system, neutrophil subsets, the microbiome, and more in refractory vasculitis [21]. Managing chronic damage is also a challenge, as once kidneys are scarred or lungs fibrosed from prior flares, current therapies cannot reverse that [40]. Thus, another future direction is regenerative medicine that is how to repair organ damage sustained during vasculitic attacks [43]. Stem cell therapies or organ‐specific growth factors might one day be adjuncts to restore function after disease control [49].

## 3 Conclusion

The paradigm shift recognizing complement as a central player in AAV has opened multiple new frontiers. We now appreciate that the “pauci‐immune” label is not synonymous with “complement‐deficient” on the contrary, complement activation is a driving force behind the necrotizing inflammation of AAV. Translating this insight into therapy (currently with avacopan and beyond) will continue to improve patient care, allowing remission with fewer steroid side effects and potentially better long‐term outcomes for patients. Yet, this progress comes with the responsibility to answer new questions: Can we prevent disease relapse without lifelong immunosuppression? What are the long‐term consequences of modulating complement in autoimmune disease? And importantly, how can lessons from AAV apply to other conditions like IgA vasculitis or cryoglobulinemia, where complement is also relevant but therapeutic trials are lagging? The coming years will likely bring a richer understanding of disease mechanisms perhaps blurring the lines between “pauciimmune” and “immune‐complex” vasculitides. The ultimate vision is precision, targeted care that induces durable remission with minimal toxicity, transforming AAV from a frequently devastating illness into a manageable, even curable condition. The Complement system, once an overlooked component, has become a beacon of both mechanistic insight and therapeutic promise in this journey of discovery.

## Author Contributions

**Kehinde Sunmboye:** conceptualization, investigation, writing – original draft, visualization, methodology, validation, software, formal analysis, project administration, resources, supervision, data curation. **Pauline Millan:** investigation, writing – review and editing, validation, supervision, resources. All authors reviewed and approved the final version of the manuscript and agree to be accountable for all aspects of the work.

## Funding

The authors received no specific funding for this work.

## Ethics Statement

The authors have nothing to report.

## Consent

The authors have nothing to report.

## Conflicts of Interest

The authors declare no conflicts of interest.
